# Assessment of Resistance Components for Improved Phenotyping of Grapevine Varieties Resistant to Downy Mildew

**DOI:** 10.3389/fpls.2019.01559

**Published:** 2019-11-27

**Authors:** Federica Bove, Luigi Bavaresco, Tito Caffi, Vittorio Rossi

**Affiliations:** ^1^Department of Sustainable Crop Production, DI.PRO.VE.S., Università Cattolica del Sacro Cuore, Piacenza, Italy; ^2^Centro di Ricerca sulla Biodiversità e sul DNA Antico, Università Cattolica del Sacro Cuore, Piacenza, Italy

**Keywords:** *Plasmopara viticola*, *Vitis vinifera*, partial resistance, *Rpv*, OIV score

## Abstract

Grapevine varieties showing partial resistance to downy mildew, caused by *Plasmopara viticola*, are a promising alternative to fungicides for disease control. Resistant varieties are obtained through breeding programs aimed at incorporating *Rpv* loci controlling the quantitative resistance into genotypes characterized by valuable agronomic and wine quality traits by mean of crossing. Traditional phenotyping methods used in these breeding programs are mostly based on the assessment of the resistance level after artificial inoculation of leaf discs in bioassays, by using the visual score proposed in the 2nd Edition of the International Organization of Vine and Wine (OIV) Descriptor List for Grape Varieties and *Vitis* species (2009). In this work, the OIV score was compared with an alternative approach, not used for the grapevine-downy mildew pathosystem so far, based on the measurement of components of resistance (RCs); 15 grapevine resistant varieties were used in comparison with the susceptible variety ‘Merlot’. OIV scores were significantly correlated with *P. viticola* infection frequency (IFR), the latent period for the downy mildew (DM) lesions to appear (LP50), and the number of sporangia produced per lesion (SPOR), so that when the OIV score increased (i.e., the resistance level increases), IFR and SPOR decreased, while LP50 increased. The relationship was linear for LP50, monomolecular for IFR and hyperbolic for SPOR. No significant correlation was found between OIV score and DM lesion size, sporangia produced per unit area of lesion, length of infectious period, and infection efficiency of the sporangia produced on DM lesions. The correlation between OIV score and area under the disease progress curve (AUDPC) calculated by using the RCs and a simulation model was significant and fit an inverse exponential function. Based on the results of this study, the measurement of the RCs to *P. viticola* in grapevine varieties by means of monocyclic, leaf disc bioassays, as well as their incorporation into a model able to simulate their effect on the polycyclic development of DM epidemics in vineyards, represents an improved method for phenotyping resistance level.

## Introduction

The ooomycete *Plasmopara viticola* originates from North America (Millardet, 1881) and is the causal agent of downy mildew (DM), one of the major diseases of *Vitis vinifera L.* worldwide. Its control still largely relies on fungicide treatments, which have environmental, social and economic impacts (Gessler et al., 2011). However, according to the Directive 2009/128/CE on the sustainable use of pesticides, the use of plant protection products has to be limited and alternative approaches to DM control are then needed (Rossi et al, 2012). The use of grapevine varieties showing partial resistance to DM represents an important tool for disease control (Töpfer et al., 2011) because it is compatible with other management options and does not have negative environmental impacts.

Wild grapevine species from North America and Asia, pertaining to the genera *Vitis* and *Muscadinia*, developed different mechanisms of resistance against *P. viticola* because of their coevolution with the pathogen (Kortekamp et al., 1997; Bellin et al., 2009; Casagrande et al., 2011; Gessler et al., 2011; Yu et al., 2012). The resistance response is conferred by quantitative trait loci (QTLs) named *Rpv* (meaning: “Resistance to *P. viticola*”); to date, 14 *Rpv* loci have been identified (Marino et al., 2003; Merdinoglu et al., 2003; Fischer et al., 2004; Wiedemann-Merdinoglu et al., 2006; Welter et al., 2007; Bellin et al., 2009; Marguerit et al., 2009; Peressotti et al., 2010; Blasi et al., 2011; Moreira et al., 2011; Di Gaspero et al., 2012; Schwander et al., 2012; Venuti et al., 2013; Ochssner et al., 2016; Zyprian et al., 2016; Sánchez-Mora et al., 2017). Depending on the *Rpv* locus and on the host genotype (Foria et al., 2018), the resistance responses to *P. viticola* infection involve different mechanisms, such as a hypersensitive response (Bellin et al., 2009; Venuti et al., 2013; Zyprian et al., 2016), callose and lignin accumulation (Dai et al., 1995; Kortekamp et al., 1997; Gindro et al., 2003), synthesis of stilbene phytoalexins (Pezet et al., 2004; Gindro et al., 2006), cell necrosis (Boso and Kassemeyer, 2008; Bellin et al., 2009; Zini et al., 2015), induction of peroxidase activity (Kortekamp et al., 1998; Toffolatti et al., 2012), and accumulation of phenolic compounds in the plant tissues surrounding the infection sites (Langcake, 1981; Alonso-Villaverde et al., 2011).

Since the discovery of the sources of resistance to *P. viticola* (Munson, 1909; Olmo, 1971), many breeding programs have been developed in order to obtain grapevine hybrids combining disease resistance genes from the wild grapevine species with the desirable agronomic and grape-quality traits carried by *V. vinifera* cultivars. From the beginning of the 20th century, several disease resistant varieties with a good level of grape quality and, in some cases, with a vinifera-like wine (Golodriga, 1978; Alleweldt, 1980; Bouquet, 1980; Kozma, 2002) have been selected and released by breeders. Some institutions are active in these breeding programs worldwide, including: Cornell University, UC Davis, and Florida AM University in the USA; several scientific institution in China (Lu and Liu, 2015); CSIRO in Australia; University of Krasnodar in Russia; JKI Gelweilerhof in Germany; INRA and IFV in France; University of Pécs in Hungary; and University of Udine, FEM, and CREA-VE in Italy (Bavaresco, 2019).

However, the use of resistant genotypes is still limited to few areas. Currently, resistant grapevine hybrids account for about 6% of the world grape-growing surface, with Kyoho (*V. labrusca* × *V. vinifera*), a Japanese table grape variety, being the most cropped one, especially in China (365,000 ha, OIV 2015 data). Resistant hybrids for wine production are mainly spread in America and Eastern Europe, specifically in Brazil (about 41,046 ha, 83% of the national viticultural surface), USA (about 11,980 ha, 5%), Moldova (about 11,656 ha, 13%), Russia (about 9,430 ha, 37%), Hungary (about 7,450 ha, 11%), Ukraine (about 3,251 ha, 6%), and Canada (about 2,680 ha, 27%) (Anderson, 2013). In the European Union (EU), the Regulation 1308/2013/EC states that resistant varieties can be grown to produce Table and PGI (Protected Geographic Indication) wines, but not PDO (Protected Denomination of Origin) wines (where only *V. vinifera* is allowed). In this respect, the situation is not uniform across the EU; for instance, Germany has classified the new disease resistant varieties as *V. vinifera*, unlike Italy and France.

The hybridization process using conventional breeding requires several years, but genetic engineering, marker assisted selection (MAS), genetic linkage maps, the availability of the *V. vinifera* genome sequence, and the knowledge of *Rpv* all help breeders in the selection of new resistant varieties. In addition to these methods, efficient phenotyping tools play a crucial role (Eibach et al., 2007; Töpfer et al., 2011; Schwander et al., 2012).

Traditional phenotyping is based on the evaluation of resistance after natural or artificial infection on leaves or leaf discs. Usually, the *in vitro* screening of partial resistance to DM is based on leaf disc bioassays (Bavaresco and Eibach, 1987; Staudt and Kassemeyer, 1995; Cadle-Davidson, 2008) and the assessment of the degree of resistance based on a visual score (from 1 = very low to 9 = very high), according to the 2nd Edition of the OIV Descriptor List for Grape Varieties and *Vitis* species (2009), i.e., the OIV 452-1 (hereafter referred to as OIV scale). In host-pathogen systems different from grape-*P. viticola* (Savary and Zadoks, 1989; Rossi et al., 1999; Rossi et al., 2000; Gordon et al., 2006; Burlakoti et al., 2010; Willocquet et al., 2011; Azzimonti et al., 2013; Schwanck et al., 2016), the measurement of resistance components (hereafter referred to as RCs) efficiently supports the evaluation of plant genotypes showing partial resistance, a type of resistance that affects several stages of the infection cycle, such as the resistance to *P. viticola*. Resistance components analysis is based on the phenotypic dissection of resistance into its components, which classically include: infection efficiency of spores, duration of latent period (i.e., the time from infection to the start of sporulation on lesions), lesion size, production of spores on lesions, and the duration of infectious period (i.e., the time a lesion continues producing spores) (Van der Plank, 1963; Zadoks, 1972; Parlevliet, 1979).

In a previous work, a resistance components analysis was conducted for 16 grapevine genotypes, some of them carrying one or more *Rpv* loci (Bove, 2018). In the present work, a comparison was made between the OIV scale and RCs with the aim of evaluating the relationships between the two phenotypic methods. 

## Materials and Methods

### Plant Material and Inoculation

The resistance response of different grapevine varieties was previously measured in a three-year study (2014 to 2016) after artificial inoculation of leaf discs in environmentally controlled conditions (Bove, 2018). Fifteen partially resistant varieties, some of them carrying one or more *Rpv* loci known to be involved in the resistance to *P. viticola*, and the *V. vinifera* ‘Merlot’, which is susceptible to downy mildew, were used (**Table 1**). Some of the resistant varieties consist of introgression lines developed in different European breeding institutes, such as the Julius-Kuehn Institute (JKI) and the Institute of Viticulture and Enology in Freiburg in Germany, and the University of Udine in collaboration with the Institute of Applied Genomics (IGA) in Italy.

**Table 1:** Grapevine varieties showing resistance to *Plasmopara viticola* used in leaf disc bioassays, their pedigree, the reference of the breeder, and *Rpv* loci.

Variety	Pedigree	Breeder	Loci
Bronner*	Merzling × Geisenheim 6494	Becker, N. (Freiburg)	*Rpv*10
Cabernet Volos*	Cabernet sauvignon × 20/3	Castellarin, S.D.; Cipriani, G.; Di Gaspero, G.; Morgante, M.; Peterlunger, E.; Testolin, R. (IGA)	*Rpv*12
Calandro*	Domina × Regent	Eibach, R.; Töpfer, R. (JKI)	*Rpv*3.1
Calardis blanc*	Geilweilerhof GA-47-42 × S.V. 39-639 KL.1	Eibach, R.; Töpfer, R. (JKI)	*Rpv*3.1, *Rpv*3.2
Felicia*	Sirius × Vidal Blanc	Eibach, R.; Töpfer, R. (JKI)	*Rpv*3
Fleurtai*	Tocai × 20/3	Castellarin, S.D.; Cipriani, G.; Di Gaspero, G.; Morgante, M.; Peterlunger, E.; Testolin, R. (IGA)	*Rpv*12
Johanniter*	Riesling Weiss × Freiburg 589-54	Zimmermann, J. (Freiburg)	*Rpv*3.1
Merlot Kanthus*	‘Merlot’ × 20/3	Castellarin, S.D.; Cipriani, G.; Di Gaspero, G.; Morgante, M.; Peterlunger, E.; Testolin, R. (IGA)	*Rpv*3
Merlot Khorus*	‘Merlot’ × 20/3	Castellarin, S.D.; Cipriani, G.; Di Gaspero, G.; Morgante, M.; Peterlunger, E.; Testolin, R. (IGA)	*Rpv*12
**Palava**	Traminer × Mueller Thurgau	Veverka, J. (Czeck Republic)	–
Reberger*	Regent × Lemberger	Eibach, R.; Töpfer, R. (JKI)	–
Regent*	Diana × Chambourchin	Alleweldt, G. (JKI)	*Rpv*3.1
**Rkatsiteli**	Unknown	-	–
Solaris*	Merzling × Geisenheim 6493	Becker, N. (Freiburg)	*Rpv*10
Villaris*	Sirius × Vidal blanc	Eibach, R.; Töpfer, R. (JKI)	*Rpv*3.1

The introgression lines (*) were developed in different European breeding institutes, such as the Julius-Kuehn Institute (JKI) and the Institute of Viticulture and Enology in Freiburg in Germany, and the University of Udine in collaboration with the Institute of Applied Genomics (IGA) in Italy (www.vivc.de).

The inoculation method is fully described in Bove (2018). In short, for each variety, fifteen fully developed leaves (specifically the fourth leaf from the shoot apex) were detached from five plants randomly selected at three growth stages (specifically at shoot growing BBCH 18, flowering BBCH 65 and fruit set BBCH 79; Lorentz et al., 1995) and immediately transported to the laboratory in a fridge at 5°C. Five leaf discs (21 mm diameter) were excised from each leaf using a cork borer. Leaf discs were repeatedly washed under tap water to remove superficial materials, dried under a laminar flow hood and finally placed, abaxial side up, in Petri dishes on filter paper wetted with 3 ml of sterile demineralized water. Each leaf disc was inoculated with four 10 µl drops of inoculum suspension containing a population of *P. v0iticola* at 5 × 10^5^ sporangia ml^−1^ concentration. The inoculum suspension was prepared from freshly (2–3-day old) sporulating DM lesions obtained by inoculating the leaves of ‘Merlot’ plants grown in pots in a greenhouse by using a bulk of sporangia produced on field-grown leaves collected from different *V. vinifera* varieties in several untreated vineyards of northern Italy. Fresh sporangia produced on these leaves were collected, diluted in sterile water, adjusted to 5 × 10^5^ sporangia ml^−1^, and immediately used for the inoculation of leaf discs. After inoculation, leaf discs were incubated at 20°C with a 12-h photoperiod.

### Phenotypic Assessment Using the OIV Scale

**Figure 1:**
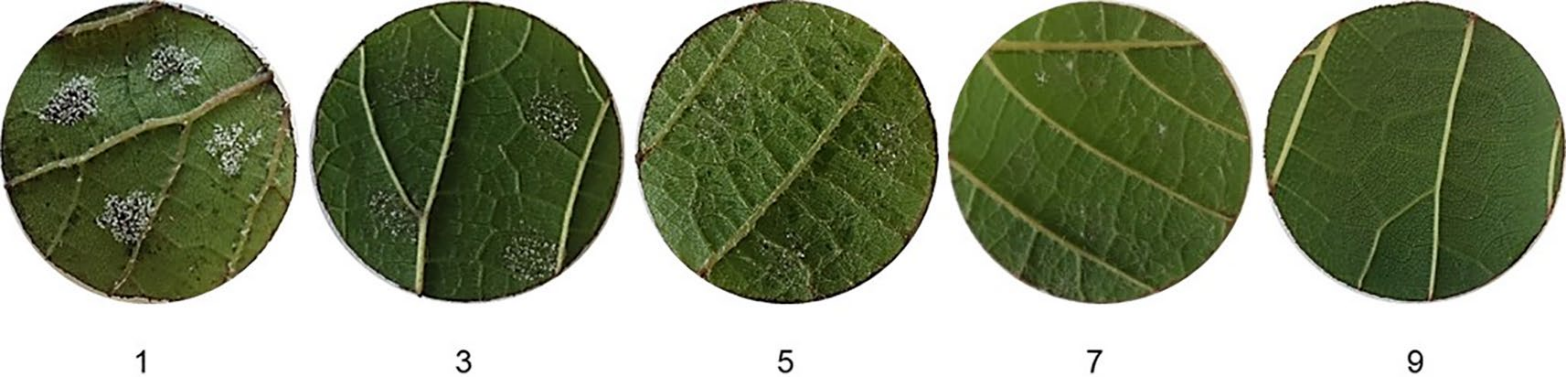
Scores assigned to grape leaf discs inoculated with *Plasmopara viticola* sporangia to assess the level of resistance based on the OIV 452-1 descriptor: 1—very little degree of resistance (dense sporulation at 100% of inoculation sites, on large lesions); 3—little (dense sporulation at 50 to 75% of inoculation sites, on large lesions); 5—medium (sparse sporulation at 50 to 75% of inoculation sites, on medium size lesions); 7—high (sparse sporulation at 25% of inoculation sites, on small size lesions); 9—very high (no sporulation). Inoculation was performed by placing on each leaf disc four 10µl drops of a suspension of 5 × 10^5^ sporangia ml^−1^.

Leaf discs were observed both visually and with the help of a microscope (at tenfold magnification), in order to observe single sporangiophores of *P. viticola* (Schwander et al., 2012), at 11 days post inoculation (dpi), and assigned to one DM resistance score based on the OIV scale (OIV, 2009), as follows: 1—very little degree of resistance to *P. viticola* (dense sporulation at 100% of inoculation sites, on large lesions); 3—little (dense sporulation at 50 to 75% of inoculation sites, on medium to large lesions; 5—medium (sparse sporulation at 50% to 75% of inoculation sites, on medium size lesions); 7—high (sparse sporulation at 25% of inoculation sites, on small size lesions); 9—very high (no sporulation) (**Figure 1**). All the assessments were made by a single expert to minimize subjectivity.

### Phenotypic Assessment Using the RCs

The following components of partial resistance were considered: i) infection frequency (IFR), ii) duration of latent period (LP50), iii) lesion size (LS), iv) number of sporangia per lesion (SPOR) and per mm^2^ of DM lesion (SPOR’), v) infectious period (IP), iv) infection efficiency of the sporangia produced on inoculation sites and re-inoculated on the susceptible variety ‘Merlot’ (INF). Methods for the measurements of RCs are described in Bove (2018). Briefly, IFR was measured at 11 dpi as the proportion of the inoculation sites showing DM sporulation over the total inoculated sites (where 0 = no inoculation sites show sporulation to 1 = all inoculation sites show sporulation). LP50 was measured in degree days (DDs, base 0°C) cumulated between the inoculation and when 50% of the inoculation sites resulting in lesions at 11 dpi showed a DM lesion. For the measurement of LS, leaf discs were photographed at 11 dpi and the area (in mm^2^) of each lesion was determined using the image analysis software Assess 2.0 (Lakhdar Lamari, APS PRESS, Saint Paul, Minnesota). SPOR was measured at 11 dpi as follows: sporangia produced on all the sporulating lesions of a leaf disc were carefully collected by using a sterile needle, suspended in 100 µl of sterile water, counted using a haemocytometer, and finally expressed as the number of sporangia per DM lesion. SPOR’ was also calculated by dividing the number of sporangia per lesion by the lesion size, and expressed as the number of sporangia per mm^2^ of DM lesion. IP was measured as the number of sporulating events on a DM lesion; after each sporulation, all the sporangiophores and sporangia were gently removed from the lesion using a sterile cotton swab, and this was repeated until the lesion no longer produced new sporangia. In order to measure INF, sporangia were collected at 11 dpi from leaf discs and then re-inoculated on new fresh leaf discs of ‘Merlot’, using the same procedure for inoculation and incubation previously described. INF was then measured as the proportion of inoculation sites showing DM sporulation on ‘Merlot’. The measurement procedures, the statistical analyses and all the details concerning the assessment of partial resistance components are described in Bove (2018).

The following RCs: IFR, LP50, SPOR and IP, were incorporated into a model able to simulate downy mildew epidemics in partially resistant varieties. The model is described in Bove (2018). In short, the model captures the main features of the grapevine downy-mildew pathosystem, i.e. primary and secondary infections, physiology and dynamics of the host (crop growth, senescence and ontogenic resistance), and development of the disease on leaves and clusters. The parameterization of the model was performed according to the available literature (Bove, 2018) and considers the main environmental variables influencing the pathosystem (rain, wetness and temperature) by using a scenario approach. The model was developed by using the STELLA^®^ software (Isee Systems, Inc, 2005). The model was operated in a favourable (i.e. not limiting) scenario for the disease, for each of the 16 varieties, by using the RCs measured in the leaf disc bioassay, so that the disease progress over time of the DM severity was simulated for each variety. The Area Under the Disease Progress Curve (AUDPC) (Campbell and Madden, 1990) was also calculated for comparing these epidemics.

### Data Analysis

In order to compare the two phenotypic methods, a correlation analysis was conducted between OIV scores, each of the seven RCs, and the AUDPC, by calculating the Pearson’s correlation coefficient, r. When correlations were significant at P ≤ 0.05, a regression analysis was conducted by fitting different equations to the data, in which the OIV score was the independent variable and the RC or the AUDPC was the dependent one. All statistical analyses were all performed by using the SPSS software version 24 (SPSS, Chicago, IL).

## Results

**Figure 2:**
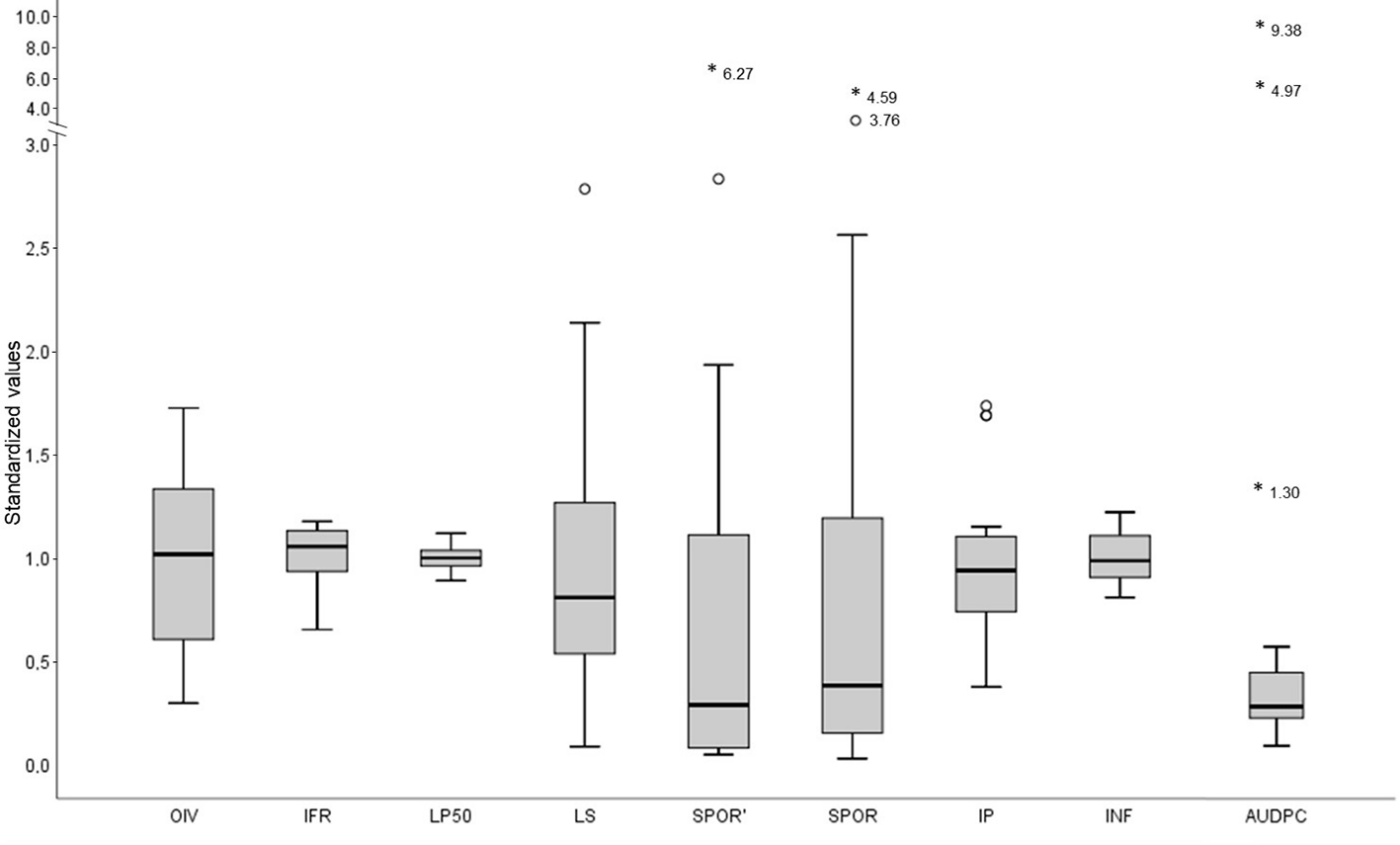
Box plots showing the distribution of OIV scores, components of partial resistance, and AUDPC in the 16 grapevine varieties. Original values of single varieties were standardized by dividing them by the average of the 16 varieties, to facilitate comparison among variables that are expressed in different units. OIV is the OIV score (average 4.6); IFR is the infection frequency of sporangia (average 0.83, in a 0-1 interval); LP50 is latent period (average 102 degree-days); LS is the lesion size (average 7.6 mm^2^); SPOR’ is the number of sporangia per mm^2^ lesion (average 344 sporangia × mm^−2^); SPOR is the number of sporangia per lesion (1,582 sporangia × lesion); IP is the infectious period (average 2.84 sporulation events); INF is the infectivity of the sporangia produced on the varieties and inoculated on the susceptible ‘Merlot’ (average 0.77 in a 0–1 interval); AUDPC is the area under the disease progress curve calculated using a simulation model (average 42,679). The box representing the AUDPC is multiplied by 10 to visually appreciate its distribution. The thick line in the boxes is the median; the lowest value in each box represents the 1st quartile (25th percentile); the top part of each box represents the 3rd quartile (75th percentile). Circles and asterisks in the graph are outliers, i.e. values that are far or very far from the rest of the values, respectively.

Results on the phenotypic assessment of partial resistance components have been shown in detail in Bove (2018). Box plots of **Figure 2** show the distribution of OIV scores, components of resistance, and AUDPC in the 16 grapevine varieties. In **Figure 2**, original values of single varieties were standardized by dividing them by the average of the 16 varieties, to facilitate comparisons among variables that are expressed in different units.

**Figure 3:**
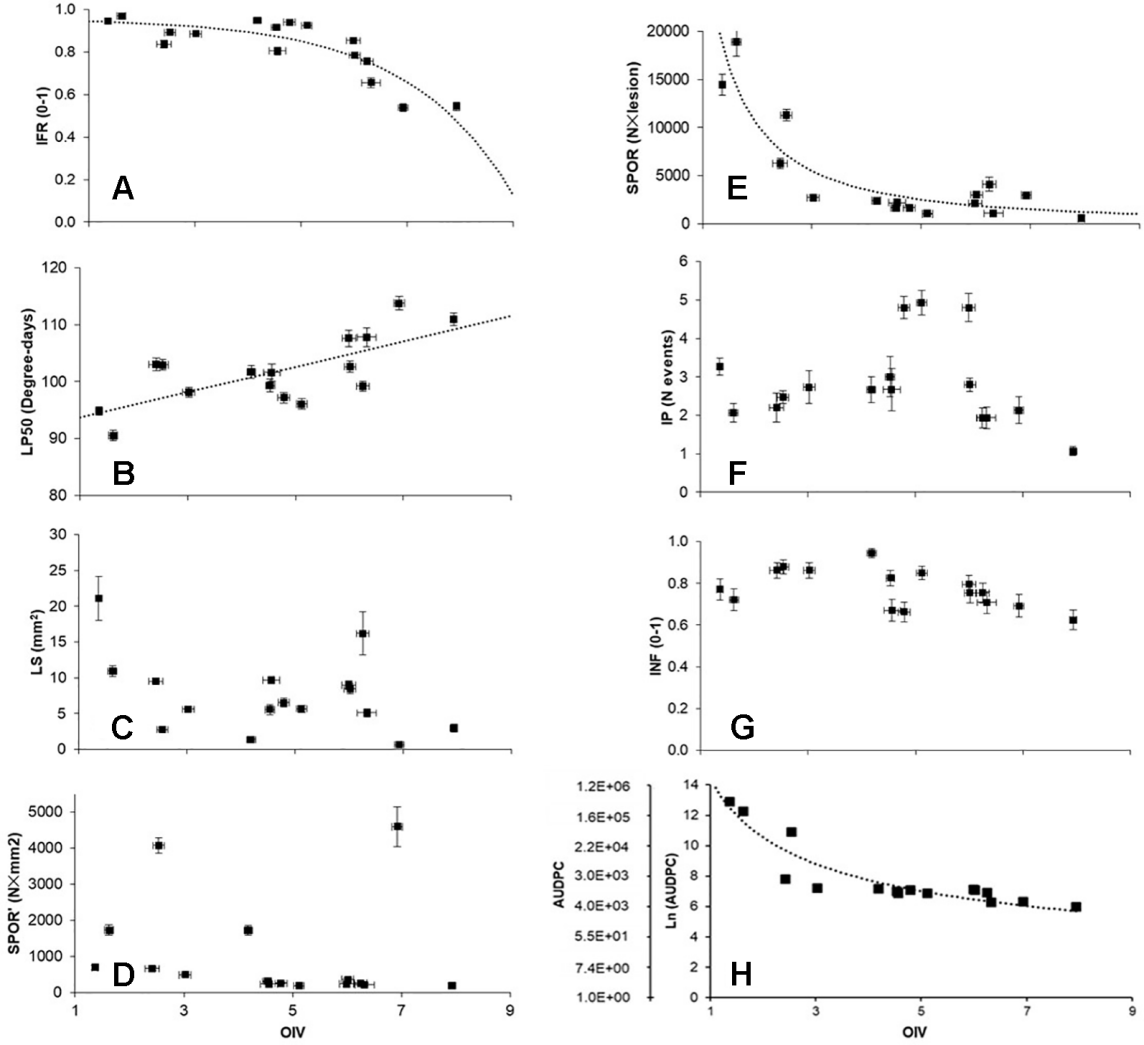
Correlations between OIV score (see Figure 1), components of partial resistance (RCs) (panels A to G) measured for 16 grapevine varieties through artificial inoculation of Plasmopara viticola sporangia in leaf discs bioassays, and the area under disease progress curve (AUDPC) calculated by using the RCs and a simulation model (panel H). RCs are: infection frequency of sporangia (IFR; 0–1; panel A); latent period (LP50; degree-days; panel B); lesion size (LS; mm2; ;panel C); number of sporangia per mm2 lesion (SPOR’; panel D); number of sporangia per lesion (SPOR; panel E); infectious period (IP; number of sporulation events; panel F); infectivity of the sporangia produced on the varieties and inoculated on the susceptible ‘Merlot’ (INF; 0–1; panel G). For significant correlations (**Table 2**), data were fit by the equations described in **Table 3** (dotted lines).

Relationships between OIV score and the seven RCs measured for the 16 grapevine varieties are shown in **Figure 3**. OIV scores were significantly correlated with IFR (r = −0.759; P = 0.001), LP50 (r = 0.710; P = 0.002), and SPOR (r = −0.763; P = 0.001), so that when the OIV score increased (i.e., the resistance level increases), IFR and SPOR decreased, while LP50 increased (**Table 2**). The relationship was linear for LP50 (**Figure 3B**), so that the latent period increased by 2.22 degree-days for each unit of the OIV score (**Table 3**, Equation 2). For the infection frequency, the relationship fit a monomolecular equation (**Table 3**, Equation 1); based on this equation, IFR decreased at very slow rate when the OIV score increased from 1 to 5, at higher rates when OIV rated 5 to 7, and at a very high rate when the OIV score was >7 (**Figure 3A**). Conversely, the relationship was hyperbolic and inversely proportional for the production of sporangia on DM lesions (**Table 3**, Equation 3); therefore, the sporulation decreased sharply when the OIV score ranged 1 to 3, and then it decreased at lower and reducing rates (**Figure 3E**).

**Table 2:** Coefficients of correlation (r) between the OIV score assigned as in **Figure 1** and the components of partial resistance (RCs) to *Plasmopara viticola* measured for 16 grapevine varieties through artificial inoculation of *P. viticola* sporangia in leaf discs bioassays and the area under disease progress curve (AUDPC) calculated by using the RCs and a simulation model.

		RCs							AUDPC
		IFR	LP50	LS	SPOR’	SPOR	IP	INF	
**OIV score**	**r**	−0.759	0.710	−0.759	−0.759	−0.759	−0.759	−0.759	−0.759
	**P-value**	0.001	0.002	0.001	0.001	0.001	0.001	0.001	0.001

RCs are: IFR, infection frequency of sporangia (0-1); LP50, latent period (degree-days); LS, lesion size (mm^2^); SPOR’, number of sporangia per mm^2^ lesion; SPOR, number of sporangia per lesion; IP, infectious period (number of sporulation events; INF, infectivity of the sporangia produced on the varieties and inoculated on the susceptible ‘Merlot’ (0–1).

**Table 3:** Parameters and statistics of the regression equations used for fitting the relationship between the OIV score assigned as in **Figure 1** (independent variable X), some components of partial resistance (RCs, dependent variable Y) to *Plasmopara viticola* measured for 16 grapevine varieties through artificial inoculation of *P. viticola* sporangia in leaf discs bioassays (specifically: IFR, infection frequency of sporangia, Equation 1; LP50, latent period, Equation 2; SPOR, number of sporangia per lesion, Equation 3) and the area under disease progress curve (AUDPC, depend variable Y) calculated by using the RCs and a simulation model (Equation 4).

Equations		Estimated parameters			R^2^
		a	b	c	
(1)	y = a − b × c^(-X)^	0.960	0.008	0.601	0.763
		*0.053* *^a^*	*0.012*	*0.109*	
(2)	y = a × X + b	2.222	91.511	–	0.504
		*0.589*	*2.921*		
(3)	y = b × X^(−a)^	1.537	29799.00	–	0.834
		*0.221*	*4662.48*		
(4)	y = b × X^(−a)^	0.447	14.393	–	0.848
		*0.047*	*0.866*		

a Standard errors of the estimated parameters.

No significant correlation was found between OIV score and LS (r = −0.393, P = 0.132), SPOR’ (r = −0.142, P = 0.601), IP (r = −0.101, P = 0.709), or INF (r = −0.470, P = 0.066) (**Table 2**).

The correlation between OIV score and AUDPC was negative and significant (r = −0.633; P < 0.001; **Table 2**). The relationship between these two variables fits an inverse exponential function, with R^2^ = 0.989 (**Table 3**, Equation 4). Specifically, all the grape varieties with an OIV score between 3 and 9 showed similar epidemic patterns, with very low AUDPC values (**Figure 3H**).

## Discussion

This paper shows a comparison between two phenotypic methods used to assess partial resistance to *P. viticola* in grapevine trough an *in vitro* bioassay with artificial inoculation of leaf discs. Leaf disc inoculation is a well-established method to obtain reliable data for assessing grape resistance to *P. viticola* and many studies have revealed its strong correlation with data from naturally or artificially infected plants in the field or in pots (Stein et al., 1985; Eibach et al., 1989; Staudt and Kassemeyer, 1995; Brown et al., 1999; Boso et al., 2006; Boso and Kassemeyer, 2008; Bellin et al., 2009).

The phenotypic response of leaves in these bioassays mainly focused on the sporulation severity, assessed by means of the OIV 452-1 (Eibach et al., 2007; Bellin et al., 2009; Deglene-Benbrahim et al., 2010; Peressotti et al., 2010; Calonnec et al., 2012; Foria et al., 2018). For instance, Deglene-Benbrahim et al. (2010) scored leaf discs based on the severity of sporulation (abundant, dense, sparse, weak or absent) and its distribution (equally distributed or in large, small or very small patches) at 6 dpi. In epidemiological terms, the assessment of sporulation by the OIV scale accounts for the infection efficiency of the sporangia in causing infection (and then in causing DM lesions), for the density of sporangiophores and sporangia on lesions, and, indirectly, for lesion size. Results of the present work show that the OIV score is correlated with the number of sporangia produced on a DM lesion (SPOR). Similarly, a strong correlation between the number of sporangia per spray-inoculated leaf disc and the OIV score was found by Calonnec et al. (2013) and the number of sporangia per unit of leaf area and a modified OIV score by Bellin et al. (2009). However, the present work shows that this relationship is nonlinear. In addition, the OIV score is not related to the number of sporangia produced per mm^2^ of DM lesion (SPOR’), which represents the real sporulation potential of *P. viticola* on leaf tissue. Therefore, the OIV scale does not represent adequately the quantitative differences in sporulation of resistant genotypes compared to a susceptible one (‘Merlot’ in this work). Concerning relevant periods of epidemics related to sporulation, the OIV score is linearly related to the time a DM lesion takes for starting to produce sporangia (i.e., the latent period, LP50), but it is not related to the time a lesion continues producing sporangia (i.e., the infectious period, IP), which is a key component for the development of polycyclic diseases in the vineyard, as DM is.

Vezzulli et al. (2018) found a significant correlation between OIV 452-1 score and DM severity on spray-inoculated leaf discs, and supplemented the OIV descriptor by introducing the disease severity score, according to OEPP/EPPO, as follows: 1, corresponding to a leaf disc with more than 40% of the area occupied by sporulation, at 6 dpi (i.e., disease severity, DS > 40%); 2, DS ranging from 30 to 40%; 3, DS 25 to 30%; 4, DS 20 to 25%; 5, DS 15 to 20%; 6, DS 10 to15%; 7, DS 5 to 10%; 8, DS 1 to 5%; 9, DS < 1%. In epidemiological terms, the assessment based on disease severity accounts for infection frequency and lesion size. In the present work, the OIV score was correlated with the IFR, which is a measure of the infection efficiency of *P. viticola* inoculum of leaves, but it is not correlated with the LS. In addition, the relationship between OIV score and IFR is not linear. Therefore, the OIV scale does not accurately reflect the ability of resistance to reduce the disease severity of DM. Finally, the OIV scale does not clearly reflect the ability of the sporangia produced on resistant plants to cause new infections, which is an important driver of DM development in natural epidemics.

The OIV 452-1 descriptor considers only some aspects of partial resistance, in a monocycle. This approach may be reductionist and does not adequately represent the ability of plant resistance to reduce the disease development under natural vineyard conditions. This is confirmed by the nonlinear relationship existing between the OIV score and the AUDPC of the varieties considered in the present work, as determined through using a simulation model for monocycle concatenation during DM epidemics. The simulation model enabled the exploration of each component of partial resistance and of its effect in slowing down epidemic development, alone or in combination with other RCs (Van der Plank, 1963). In a previous work (Bove, 2018), the infection frequency showed the strongest effect in reducing the disease development because it affects both primary and secondary infections. The number of sporangia produced per lesion also had a strong effect in slowing the disease progress because the higher number of propagules produced per lesion caused a higher basic infection rate (R_c_) (Zadoks and Schein, 1979). Therefore, resistant varieties showing low IFR and SPOR in monocyclic bioassay have great potential for slowing down DM epidemics in the vineyard. Varieties showing a shorter IP compared to susceptible ones also reduce epidemic development, because the number of secondary infection cycles originating from a lesion is lower. Similarly, the varieties showing a longer latent period slow down DM epidemics because the time at which they start producing sporangia for new infections is delayed.

The low accuracy of the OIV scale in representing the real effect of a resistant variety on slowing down DM epidemics in the vineyard is also confirmed by some contrasting results of previous works. In some works, a relationship was found between resistance levels from visual observation of leaf disc in bioassays and from field assessments (Sotolář, 2007; Boso et al., 2014; Zyprian et al., 2016; Buonassisi et al., 2018; Foria et al., 2018; Vezzulli et al., 2018). In other works, no relationship was found and this was imputed to the inoculation of a single *P. viticola* strain, which may be not representative of a field population (Cadle-Davidson, 2008), or to high inoculum concentration and optimal environmental conditions of the leaf discs bioassay, which may lead to an underestimation of the resistance level with the OIV scale (Calonnec et al., 2013). In addition (or as an alternative) to these argumentations, the inconsistent relationship between the resistance level of a variety in the bioassay assessed through the OIV scale and in the vineyard may be due to the fact that monocyclic experiments (i.e., leaf disc bioassays) and the OIV scale do not account for all the effects of partial resistance on each infection cycle and in the concatenation of infection cycles during the season as well. It might be also considered that in this study a mixture of isolates collected from different varieties in multiple vineyards were used for artificial inoculation of different resistant grapevine accessions carrying different resistant loci. It is known that some DM isolates are more aggressive than others (Delmotte et al., 2014), and each specific isolate/accession pair may have a unique scenario in term of disease severity and RCs.

Based on the results of this study, the measurement of the components of partial resistance to *P. viticola* in grapevine varieties by means of monocyclic leaf disc bioassays, as well as their incorporation into a model able to simulate their effect on the polycyclic development of DM epidemics in vineyards represents an improved method for phenotyping resistance level. In addition, the measurement of resistance components is a quantitative method, which is not influenced by the subjective bias of qualitative methods, like the OIV descriptor, and this would reduce bias of phenotyping outcomes. Therefore, resistance component analysis meets all the criteria for efficient phenotyping tools needed to grapevine breeders for the selection of new resistant genotypes (Töpfer et al., 2011).

## Data Availability Statement

Datasets are available on request: The raw data supporting the conclusions of this manuscript will be made available by the authors, without undue reservation, to any qualified researcher.

## Ethics Statement

The authors declare that the present manuscript complies with the Ethical Rules of good scientific practice applicable for this Journal. The research does not involve humans or animals.

## Author Contributions

FB and VR mainly contributed to the conception and the design of the study. TC contributed to the analysis of results. FB and VR wrote the first draft of the manuscript. TC and LB contributed to the critical analysis of the manuscript. All authors contributed to manuscript revision, read, and approved the submitted version.

## Funding

This research was supported by the European Community’s Seventh Framework Programme (FP7-2007-2013) under grant agreement no. 311775 project INNOVINE.

## Conflict of Interest

The authors declare that the research was conducted in the absence of any commercial or financial relationships that could be construed as a potential conflict of interest.
